# Draft Genome Sequence and Annotation of *Oribacterium* sp. Strain C9, Isolated from a Cattle Rumen

**DOI:** 10.1128/MRA.01562-18

**Published:** 2019-03-28

**Authors:** Seungha Kang, Stuart Denman, Chris McSweeney

**Affiliations:** a CSIRO Agriculture and Food, Queensland Bioscience Precinct, St. Lucia, Queensland, Australia; University of California, Riverside

## Abstract

We report the 3.7-Mb genome sequence of *Oribacterium* sp. strain C9, isolated from the rumen of a steer grazing on Rhodes grass in Rockhampton, Queensland, Australia. This draft genome consists of 3,720,024 bp with a 42.8% G+C content, 3,130 predicted coding DNA sequences (CDSs), and 67 RNAs.

## Abstract (precis)

We report the 3.7-Mb genome sequence of *Oribacterium* sp. strain C9, isolated from the rumen of a steer grazing on Rhodes grass in Rockhampton, Queensland, Australia.

## ANNOUNCEMENT

The genus *Oribacterium* was first proposed by Jean-Philippe Carlier (1). The initial representative of this genus was isolated from sinus pus of a child, and a few other *Oribacterium* bacteria have been isolated from the human oral cavity (2). Here, we report on *Oribacterium* sp. strain C9, isolated from rumen digesta of cattle grazing on Rhodes grass (Chloris gayana) in Australia (Rockhampton, Queensland), using an anaerobic *in vitro* culture with 0.8% yeast extract. DNA sequencing was performed at Macrogen (South Korea) on a HiSeq 2500 instrument (Illumina, South Korea) (2 × 100-bp paired-end sequencing) to produce 58,649,712 total reads with 15× coverage. Raw reads were trimmed with Trimmomatic version 0.32 (parameters used, phred + 64 quality scores) (3) followed by *de novo* assembly using the SPAdes version 3.6.0 algorithm with default settings in KBase (4). We obtained 84 contigs (the largest one being 322,617 bp) with a total length of 3,720,024 bp (using only contigs larger than 500 bp), a G+C content of 42.8%, and an *N*_50_ value of 121,148 bp defined by the quality assessment tool for genome assemblies (QUAST) version 4 (5). The draft genome annotated by the NCBI Prokaryotic Genome Annotation Pipeline (https://www.ncbi.nlm.nih.gov/genome/annotation_prok) (6) predicted 3,252 genes, including 3,185 coding DNA sequences (CDSs), 10 rRNAs (6 16S and 4 23S), 54 tRNAs, and 3 noncoding RNAs (ncRNAs).

The 16S rRNA gene sequence was found to be 95% identical to that of Oribacterium parvum strain ACB1^T^ (2) and Oribacterium sinus F0268 (1) using BLAST 2 sequences (https://blast.ncbi.nlm.nih.gov/). The average nucleotide identity (ANI) was calculated using the algorithm described by Yoon et al. (7) with the EzBioCloud portal. The draft genome of strain C9 was 82.38%, 77.85%, 76.76%, 76.51%, 76.47%, 68.38%, and 68.18% identical to that of *Oribacterium* sp. strains P6A1 (GenBank accession number JNKO00000000), WCC10 (FOPH00000000), NK2B42 (AUJX00000000), FC2011 (JNJM00000000), and KHPX15 (FNRG00000000) (from the Hungate1000 project [8]), *O. parvum* strain ACB1^T^ (AFZC00000000), and O*. sinus* F0268 (ACKX00000000), respectively.

The digital DNA-DNA hybridization (dDDH) was determined using the Genome-to-Genome Distance Calculator (GGDC) version 2.1 with default settings (9). The estimated dDDH values between strain C9 and the reference genomes *Oribacterium* sp. strains P6A1, WCC10, NK2B42, FC2011, and KHPX15, *O. sinus* F0268, and *O. parvum* strain ACB1^T^ are 28.4%, 24.9%, 24.4%, 24%, 24.1%, 21.1%, and 26.1%, respectively. Having both ANI values lower than 95% and dDDH values lower than 70% indicated that isolated strain C9 is a species distinct from the other isolates.

Utilizing all functional annotations from the Carbohydrate-Active enZYmes Database (CAZy) (http://www.cazy.org/) (10) and dbCAN (http://csbl.bmb.uga.edu/dbCAN/) (11), we identified 40 sequences encoding potential glycosyl hydrolases (GH), 33 glycosyl transferases (GT), and 9 carbohydrate esterases (CE). Two pectin-degrading enzymes were identified in the genome. The polygalacturonase (EC 3.2.1.15, glycoside hydrolase family 28 [GH28]) is known as pectin depolymerase, which can hydrolyze the alpha-1,4 glycosidic bonds between galacturonic acid residues and the pectinesterase (EC 3.1.1.11, carbohydrate esterase family 8 [CE8]) and can facilitate plant cell wall modification and subsequent breakdown. Strain C9 is capable of degrading pectin from plant cell walls in the rumen environment. The genome information presented here will inform further studies investigating the mechanism by which complex carbohydrates are degraded.

### Data availability.

The draft genome sequence for *Oribacterium* sp. strain C9 has been deposited in DDBJ/EMBL/GenBank under the accession number MUHW00000000, BioProject number PRJNA362832, and BioSample number SAMN06249574. The genomic raw sequencing reads are available in the Sequence Read Archive (SRA) database under accession number SRR8449972.
